# Epstein–Barr Virus Mucocutaneous Ulcer: An Unexpected Diagnosis of a New Entity

**DOI:** 10.1155/2021/9989756

**Published:** 2021-05-10

**Authors:** William Howden, Tony Kuo

**Affiliations:** ^1^Discipline of Surgery, University of Sydney, Sydney, Australia; ^2^Department of Otolaryngology Head and Neck Surgery, Gosford Hospital, Gosford, Australia

## Abstract

Epstein–Barr virus mucocutaneous ulcer (EBVMCU) is a new entity, only recently included in World Health Organisation classification of lymphoid neoplasms. Almost all cases described to date have been in patients with a predisposing risk factor of immunosuppression. This case presents a 21-year-old male admitted with tonsillitis and no overt immunosuppression, who is subsequently diagnosed with EBVMCU of likely iatrogenic origin.

## 1. Introduction

Epstein–Barr virus mucocutaneous ulcer (EBVMCU) is a rare, underdiagnosed, and newly recognised clinical entity, affecting the sino-oral cavities, gastrointestinal tract, and skin. EBVMCU is a provisional entity in the 2016 Update of World Health Organisation classification of lymphoid neoplasms. In the literature to date, a vast majority of cases have been described in immunosuppressed patients, usually as a result of autoimmune, transplantation, or haematological malignancy therapy. This report examines the course and treatment of an otherwise well young male presenting clinically with tonsillitis, who was subsequently diagnosed with EBVMCU on further testing.

## 2. Case Presentation

A 23-year-old male presented to a district general hospital with a 1-day history of progressive odynophagia, blood-streaked sputum, unilateral neck pain and swelling, and associated subjective fevers. He had no notable medical history including autoimmune disorders or cancer and denied any regular medications. He had smoked 10–20 cigarettes per day for the past 5 years.

Upon examination, he was afebrile on admission, with left-sided tender level II/III lymph nodes and normal range of motion of his neck. On direct and nasoendoscopic examination of his oropharynx, he was noted to have grade II bilaterally erythematous palatine tonsils, with a white coating to the right inferior pole with mildly erythematous. On flexible nasoendoscopy, he had symmetrical and nonoedematous arytenoids and epiglottis. He had no trismus, palatal oedema or petechiae, uvula deviation, or periorbital oedema. He was not examined for splenomegaly.

His bloods revealed a lymphocytosis of 9.0 × 10^9^/L and a C-reactive protein (CRP) of 10 mg/L. A serum monospot, added retrospectively to admission bloods, was positive.

A computed tomography (CT) scan of his neck demonstrated a 7 × 8 × 7 mm collection posterior to his left palatine tonsil. There was no evidence of deep neck space involvement. This was not drained due to the absence of clinical features of peritonsillar abscess. The patient was originally discharged with analgesia and advise. However, he represented the following day with worsening pain.

He was admitted to the ward and treated with IV amoxicillin and clavulanic acid, analgesia, and IV dexamethasone.

On day 3 of medical management, he began to spike fevers to 39°C and had ongoing, severe odynophagia. His lymphocytes increased to 11.9 × 10^9^/L. Due to radiological collection and worsening pain, an emergency tonsillectomy was subsequently performed, and the excisional biopsy was sent for histopathological analysis.

Following tonsillectomy, the patient remained in considerable postoperative pain, for which he was prescribed further dexamethasone. He was discharged on postoperative day 2.

**Figure 1:**
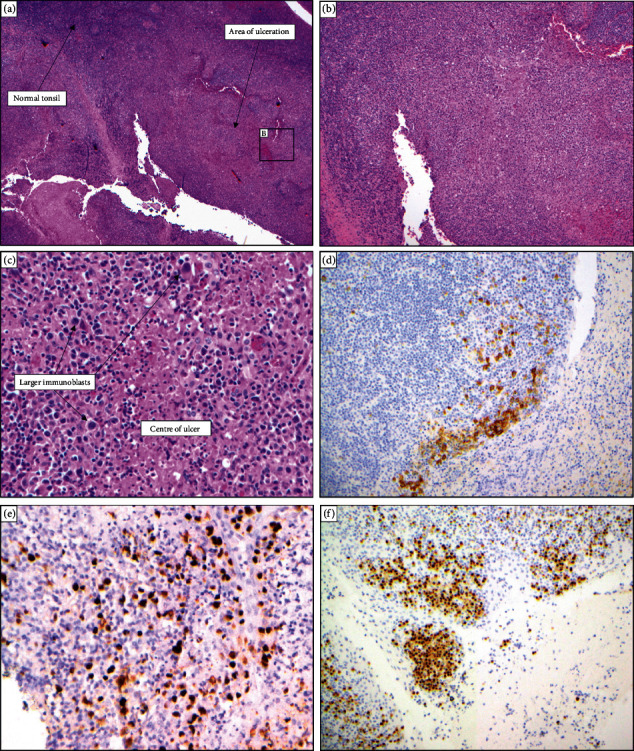
Histopathology demonstrating the normal tonsillar tissue with areas of deep ulceration at 4x (a) and 10x (b) magnification. At 40x magnification, the large immunoblasts and the occasional Reed–Sternberg-like cells are demonstrated in the centre of the ulcer (c). The larger immunoblasts demonstrated positive staining for CD-30 (d), EBERish (e), and MUM-1 (f).

Histopathology (Figure 1) demonstrated several shallow, sharply circumscribed ulcers with the ulcer base showing some larger, atypical cells including smudged cells with variable nuclear size and shape. Immunohistochemistry demonstrated positivity for CD-20, EBER, CD-30, MUM-1, and PAX-5. The slides were sent for expert opinion, and the diagnosis of EBV mucocutaneous ulcers confirmed.

At 6 months, there was no evidence of disease recurrence.

## 3. Discussion

Epstein–Barr virus (EBV) mucocutaneous ulcer (MCU) is a rare but underdiagnosed condition, presenting as solitary, sharply demarcated ulcerations of the oral cavities, gastrointestinal tract, and skin [1]. The disease is a relatively new clinicopathological entity since its inclusion in the 2016 World Health Organisation (WHO) classification of lymphoid neoplasms [2]. Due to its many histopathological similarities, it has previously been undifferentiated from the EBV-associated lymphoproliferative disorders (LPDs), in particular EBV + diffuse large B-cell lymphoma (EBV + DLBL). This distinction was made and is of particular importance given the drastic differences in outcomes favouring conservative management in EBVMCU in contrast to EBV + DLBCL [3, 4]. These characteristics are summarised in Table 1.

The median age of patients with EBVMCU is 68.5 years, with a female predilection (60%) [3]. A vast majority (90%) of cases are unifocal, most commonly involving in the mouth/oral cavity (58%), gastrointestinal tract (20%), skin (19%), and rarely the sinonasal cavity (3%).

Almost all cases of EBVMCU reported to date possess at least one of the WHO-defined, predisposing risk factors of immunosuppression (medication-induced, age-related immunosenescence, and primary and acquired immunodeficiency disorders) [3], which is proposed to contribute significantly to its pathogenesis.

The pathogenesis of EBVMCU is not fully established. After initial infection at an early age, EBV will continue to infect B cells of most adults. Through complex mechanisms, the virus then elicits the transformation and proliferation of B cells. Physiologically, this characteristic propensity for of EBV to induce the proliferation of B cells is balanced by complex immunologic interactions which are effective to maintain EBV-infected cells at very low levels in immunocompetent individuals [5]. It is speculated either age or medication reduces immune surveillance to a level which is only just sufficient to maintain the virus in its dormant state [6]. Further exposure to an immunomodulating factor is then thought to tip the delicate immunological balance, allowing localised EBV-driven lymphoproliferation [5]. Locations where EBV + B cells are abundant such as Waldeyer's ring may be particularly prone to this disequilibrium [4, 6].

There remains no distinct diagnostic criteria for EBVMCU and therefore requires correlation of clinical, histopathological, and immunophenotypic findings. Patients generally present with localised, well-circumscribed superficial ulcerations, with absence of a mass lesion. Serum generally demonstrates a lymphocytosis and evidence of EBV infection. Polymerase chain reaction (PCR) quantification of EBV-DNA in peripheral blood may also be useful, and the diagnosis of EBVMCU is questioned if high titres are isolated [7].

Microscopically, the superficial mucocutaneous lesions demonstrate a well-demarcated base with infiltration of inflammatory cells, in particular reactive T lymphocytes. Histologically, the monoclonal EBVMCU immunoblasts stain positive for CD-20, CD-30, EBER-1, MUM-1, OCT-2, and PAX-5. There is variability in staining for BCL-6, CD-15, CD-45, and CD-79a. Usually, atypical Reed–Sternberg-like cells demonstrating CD-15 and CD-30 coexpression while retaining PAX-5 positivity will be present in the polymorphic infiltrate. The dense reactive lymphocytic infiltrates on the periphery of the lesion are usually rich in CD3+ T cells [1–4].

The distinction between EBVMCU and EBV + DLBCL is vital in order to prevent exposure of these patients from unnecessary exposure to chemotherapeutic agents. Management of EBVMCU overwhelmingly favours conservative management strategies. In the age-related population, EBVMCU generally takes a self-limiting, indolent course (96.6% spontaneous remission), while in those iatrogenically suppressed, a reduction in immunosuppression is usually sufficient (94.1%) to induce remission [5, 6]. Importantly, the remainder can represent a progressive and debilitating condition, necessitating aggressive medical therapy or excision [8].

The initial presentation was diagnosed and treated as a presumed viral tonsillitis with bacterial superinfection. The patient discussed in the above report did not have any of the WHO-defined risk factors for EBVMCU. Although he had a moderate smoking history, the patient was otherwise young, fit, and healthy. Following histopathological diagnosis, the patient was counselled and referred for HIV screening and immunoglobulin analysis. The patient did not follow-up on this referral. However, his pretest probability was otherwise low. He had no other source of immunosuppression other than 3 days of inpatient treatment with dexamethasone for severe pharyngitis. It is possible that the patient had EBV + pharyngitis, with the delicate immunomodulatory balance tipped in favour of EBV by this iatrogenic immunosuppression. The use of short courses of corticosteroids is recommended by current Australian guidelines for pharyngitis unresponsive to simple analgesia [9] and is supported by high quality evidence [10]. However, the decision should be balanced against its potential for immunosuppression.

## 4. Conclusion

EBVMCU is a rare and newly described disorder. Given that the condition can masquerade as conventional tonsillitis, it may be commonly overlooked or misdiagnosed. It should be included in the differential for severe tonsillitis, particularly in those unresponsive to conventional medical management. The use of dexamethasone in tonsillitis should be a carefully considered decision made in conjunction with a serological testing.

## Figures and Tables

**Table 1:** Differentiating characteristics of EBV +ve DLBCL and EBVMCU.

	EBV +ve DLBCL	EBVMCU
Population	Usually elderly patients	Usually age-related immune somnolence or young patients with iatrogenic immunosuppression
Macroscopic	Usually associated with mass lesion	Sharply demarcated ulcers
Histopathology	Mixed inflammatory infiltrate of lymphocytes, plasma cells, histiocytes, and eosinophils	Mixed inflammatory infiltrate of lymphocytes, plasma cells, histiocytes, and eosinophils
	EBV +ve large cells only	Band of small T cells at the base of ulcer
		EBV +ve in variety of cell sizes
Growth	Aggressive	Limited
Prognosis	Poor	Favourable
Treatment	Chemoradiotherapy, surgical	Usually conservative

## Data Availability

The data used to support the findings of this study are available from the corresponding author upon request.

## Additional Points

*Summary*. (i) Epstein–Barr Virus (EBV) mucocutaneous ulcer (MCU) is a newly described, rare but underdiagnosed condition. (ii) The condition has previously been reported only in immunosuppressed individuals. (iii) EBVMCU can present as a mimic of conventional tonsillitis. (iv) EBVMCU can occur as a result of acute steroid administration in an otherwise nonimmunosuppressed population. (v) In EBV +ve tonsillitis patients, the use of dexamethasone and other immunosuppressive agents should be carefully a considered decision. (vi) The use of EBV serology can aid in this decision-making.
